# Perinatal mental health among South Asian immigrant women in Canada: A scoping review protocol

**DOI:** 10.1371/journal.pone.0354152

**Published:** 2026-07-20

**Authors:** Bibi Hajira, Kimberley Jackson, Roula Kteily Hawa, Anam Shahil-Feroz

**Affiliations:** 1 Arthur Labatt Family School of Nursing, Western University, London, Ontario, Canada; 2 Family Studies and Human Development, Western University, London, Ontario, Canada; Institute of Public Health from Guanajuato State, MEXICO

## Abstract

Perinatal mental health concerns are common and may be heightened among immigrant populations because of migration-related stressors, social isolation, stigma, language barriers, and limited access to culturally safe care. Although previous reviews have examined perinatal mental health among immigrants broadly, evidence focused specifically on South Asian immigrant women in Canada across pregnancy and the postpartum period has not been comprehensively mapped. South Asian women represent one of the largest and fastest-growing immigrant populations in Canada and may experience unique cultural, familial, linguistic, and migration-related factors that shape perinatal mental health experiences and access to care. This scoping review will map the extent, range, and nature of evidence on perinatal mental health among South Asian immigrant women in Canada by examining reported mental health outcomes, associated risk and protective factors, help-seeking experiences, and barriers and facilitators to accessing support services, and identify gaps to inform future research, practice, and policy. This protocol was developed in accordance with Joanna Briggs Institute guidance for scoping review protocols and reported using the PRISMA-P checklist, where applicable (see S2 Checklist). The completed scoping review will be reported according to the PRISMA-ScR checklist. MEDLINE, Embase, PsycINFO, CINAHL, and Scopus will be searched for literature published from January 2000 to the date of the final search. Grey literature will be identified through targeted searches of government, public health, professional, and community organization websites. Eligible sources will include qualitative, quantitative, mixed-methods, and relevant grey literature addressing perinatal mental health during pregnancy and up to 12 months postpartum among South Asian immigrant women residing in Canada. Inclusion criteria are immigrant women of South Asian origin from counties such as India, Pakistan, Bangladesh, Sri Lanka, Afghanistan, Nepal, Bhutan, and Maldives residing in Canada during the perinatal period. Two reviewers will independently screen titles, abstracts with disagreements resolved through discussion or third-reviewer adjudication. Data will be extracted using a standardized charting form and summarized using descriptive mapping and narrative synthesis. Protocols and planned studies will be charted separately from completed studies. Consistent with scoping review methodology, formal exclusion of studies based on methodological quality will not be undertaken; however, study characteristics and reported methodological limitations will be charted to support interpretation of the evidence base. Ethics approval is not required because the review will use publicly available sources only. Findings will be disseminated through a dissertation, peer-reviewed publication, and conference presentations. The review will provide a comprehensive overview of existing evidence and knowledge gaps to support culturally responsive perinatal mental health research, policy development, and healthcare practice for South Asian immigrant women in Canada. This protocol has been preregistered on the Open Science Framework:10.17605/OSF.IO/FZN4S.

## Introduction

Perinatal mental health concerns, including depression, anxiety, and other psychiatric disorders, remain a major public health challenge worldwide, affecting approximately 10% of women during pregnancy and 13% in the first year postpartum [1,2]. These concerns can have consequences beyond the individual, influencing infant development, family wellbeing, and broader social outcomes [3].

In Canada, national survey data reported that 23% of mothers who recently gave birth experienced symptoms consistent with postpartum depression or an anxiety disorder [4], and only 51% of postpartum women described their mental health as positive [5]. Population-based surveillance and estimates vary across provinces and territories due to differences in measurement tools, case definitions, timing of assessment, and populations sampled [4,5]. Ontario data indicate that between 2007 and 2021, 22–28% of perinatal individuals accessed mental health services annually, with prevalence rates estimated at 10–15% during pregnancy and 17–21% in the postpartum period [6]. Available evidence further suggests that the burden may be higher among women from diverse ethnocultural backgrounds, including recent immigrants [4,7].

The relationship between immigration and perinatal mental health is complex and influenced by multiple interacting factors. Conceptually, migration can be understood as a social and cultural transition that may affect psychological wellbeing through processes of acculturation, identity negotiation, social integration, and adaptation to new social environments. During the perinatal period, these migration-related experiences intersect with changing family roles, cultural expectations surrounding motherhood, and healthcare encounters, creating unique contexts that may shape mental health outcomes and help-seeking behaviours among immigrant women [8–14].

Canada is home to more than 2 million South Asian Canadians, representing approximately 7% of the national population [15]. South Asian immigrant women may face heightened vulnerability to perinatal mental health challenges due to migration- and settlement-related stressors, cultural stigma, language barriers, discrimination, and limited social support networks [9–13]. In addition to these broader immigrant experiences, South Asian women may encounter culturally specific influences, including collectivist family structures, expectations regarding motherhood and caregiving, gender norms, intergenerational family dynamics, culturally shaped understandings of mental health, and stigma associated with emotional distress or professional help-seeking. These contextual factors may influence symptom recognition, disclosure, coping strategies, and access to support in ways that differ from other immigrant populations. However, this population remains understudied in Canadian perinatal mental health research. A key limitation in the existing literature is the tendency to aggregate immigrants from diverse backgrounds, which can obscure culturally and socially specific contexts relevant to South Asian communities [9–14].

Existing evidence highlights several factors that may shape perinatal mental health and access to care among South Asian immigrant women in Canada, including social isolation, reduced informal supports, financial and employment precarity, and gendered expectations related to motherhood roles [8–11]. Barriers commonly reported across immigrant populations, such as stigma and fears about disclosure, limited mental health literacy, language barriers and inadequate interpretation supports, lack of culturally safe and linguistically appropriate services, and system-navigation challenges, may operate in distinct ways for South Asian families depending on migration pathways, community supports, and family structures [8, 12]. Provider-focused work similarly suggests gaps in detection and follow-up; for example, Ganann et al. [13] reported that standard postpartum depression assessments may miss women whose symptoms emerge later and that service provision may be inconsistent despite referral mechanisms. In addition, Western healthcare approaches may not align with the needs and help-seeking practices of South Asian immigrant families, contributing to unmet needs and feelings of alienation [14]. Although previous studies have identified important risk factors and barriers to care, the existing evidence remains fragmented. Research varies considerably in study design, populations included, mental health outcomes examined, and methods of assessment, making comparisons across studies challenging. Furthermore, many studies focus on immigrant populations broadly, provide limited subgroup analyses for South Asian women, or emphasize postpartum depression while overlooking other dimensions of perinatal mental health such as anxiety, distress, trauma, coping, resilience, and wellbeing. These methodological differences and gaps limit understanding of how South Asian immigrant women are represented within the Canadian evidence base and where important knowledge gaps remain.

Previous systematic and scoping reviews have examined perinatal mental health among immigrant women in Canada [8,11], Although Khan’s review addressed postpartum depression and mental healthcare access among migrant South Asian women in Canada, it was limited in scope, predates recent evidence, and focused primarily on postpartum depression rather than the broader perinatal period and wider mental health outcomes. The proposed scoping review will include pregnancy through 12 months postpartum, broader mental health outcomes including anxiety, stress, trauma, distress, coping, resilience, and wellbeing, and will map how South Asian subgroups, immigration-related variables, and service-access factors are represented in Canadian evidence. This is important because “South Asian” encompasses substantial diversity in cultural, religious, linguistic, and socioeconomic backgrounds, as well as differences by country of origin, including India, Pakistan, Bangladesh, Sri Lanka, Afghanistan, Nepal, Bhutan, and Maldives, and migration circumstances. Accordingly, this scoping review will not assume homogeneity; instead, it will examine how South Asian immigrant women are defined and represented in Canadian perinatal mental health research and whether factors such as language, migration status, religion, extended family dynamics, gender norms, informal support systems, and longer-term postpartum outcomes are considered or overlooked.

The objective of this scoping review is to map and synthesize the existing evidence on perinatal mental health among South Asian immigrant women in Canada, across pregnancy and up to one year postpartum. The review will characterize the scope and nature of the literature, including studied outcomes, populations represented or missing, assessment tools used, and reported barriers and facilitators to care. To our knowledge, no recent scoping reviews have comprehensively mapped the Canadian literature across the full perinatal period while specifically examining the diversity of South Asian immigrant populations, the range of mental health outcomes investigated, and the contextual factors shaping experiences and service access. By identifying evidence gaps, this review aims to inform future research priorities, support culturally responsive clinical practice, and guide policies to improve access to appropriate perinatal mental health services for South Asian immigrant women in Canada. The review will therefore provide an updated and comprehensive evidence map to inform research, policy, and practice while identifying priorities for future investigation.

## Methods

This protocol was developed using the JBI Manual for Evidence Synthesis guidance for scoping reviews [16] and relevant items from PRISMA-P. The completed review will be reported according to PRISMA-ScR [17]. This protocol has been preregistered on the Open Science Framework (OSF; DOI: https://doi.org/10.1146/annurev-clinpsy-050212-185612).

### Review question

This scoping review asks: What is currently known about perinatal mental health among South Asian immigrant women in Canada?

For the purpose of this review, South Asian immigrant women are defined as women residing in Canada who were born in South Asian countries (India, Pakistan, Bangladesh, Sri Lanka, Afghanistan, Nepal, Bhutan, or Maldives) and who immigrated through any migration pathway, including permanent residency, refugee or asylum status, temporary residence, international study, or work permits. Perinatal mental health is defined as mental health and wellbeing during pregnancy and up to 12 months postpartum, including depression, anxiety, stress, trauma, psychological distress, coping, resilience, and emotional wellbeing.

### Eligibility criteria

For the eligibility criteria within this scoping review, we are using the Participants, Concept, Context (PCC) framework as defined by the Joanna Briggs Institute (JBI) [16]. For a complete list of inclusion and exclusion criteria, see (Table 1).

**Table 1:** Inclusion and exclusion criteria.

PCC	Included	Excluded
**Participants**	South Asian immigrant women residing in Canada who experienced pregnancy or up to 12 months postpartum. South Asian countries include India, Pakistan, Bangladesh, Sri Lanka, Afghanistan, Nepal, Bhutan, and Maldives. Provider-only studies will be eligible only when findings specifically address perinatal mental health, care access, service delivery, or support needs of South Asian immigrant women in Canada.	Non-immigrant women; populations not identifiable as South Asian; studies where South Asian immigrant women cannot be disaggregated; Provider studies addressing immigrant women generally without disaggregated South Asian-specific findings will be excluded.
**Concept**	Perinatal mental health, including depression, anxiety, trauma, stress, coping, resilience, psychological distress, emotional wellbeing, help-seeking, service access, barriers, facilitators, and interventions.	Studies focused only on physical health or non-perinatal outcomes without mental health relevance
**Context**	Canada; pregnancy through 12 months postpartum.	Studies conducted outside Canada or outside the perinatal period.
**Article type**	Peer-reviewed empirical qualitative, quantitative, and mixed-methods studies; relevant grey literature; and protocols of ongoing or planned studies, charted separately.	Editorials, commentaries, opinion pieces, letters, blogs, and sources without primary data or a study plan. Reviews will be used only for reference-list screening.
**Date**	January 2000 to the date of the final search.	Publications before 2000.
**Language**	Full-text sources available in English.	Sources without sufficient English information for screening or extraction.

### Participants

The participants for this review will include South Asian immigrant women who have migrated to Canada from South Asian countries (India, Pakistan, Bangladesh, Sri Lanka, Afghanistan, Nepal, Bhutan and Maldives [18,19] and have experienced pregnancy and/or the postpartum period. Studies must clearly identify participants as South Asian immigrants residing in Canada; studies that do not specify the country of origin or migration status will be excluded. For this scoping review, the perinatal period is defined as pregnancy through 12 months postpartum, and studies will be included if they examine mental health during this timeframe [2] among South Asian immigrant women in Canada. Studies with mixed populations will be included only if data for South Asian immigrant women in Canada are reported separately or can be clearly extracted. Immigrant status will include foreign-born permanent residents, refugees, asylum seekers, temporary residents, international students, and other newcomer groups where reported.. Because these immigration categories may represent diverse migration experiences and settlement contexts, immigration-related characteristics (e.g., refugee status, family sponsorship, economic migration, temporary residence, length of residence in Canada, and migration pathway) will be charted and reported separately where available. This approach will allow heterogeneity across immigrant groups to be examined during data synthesis and interpretation.

### Concept

This review focuses on perinatal mental health, encompassing depression, anxiety, trauma, stress, coping, resilience, psychological distress, emotional wellbeing, maternal mental health, and related outcomes occurring during pregnancy and up to one year postpartum [20]. Both antenatal (during pregnancy) and postnatal (after birth) mental health are included. Studies assessing the prevalence, risk factors, barriers, facilitators, or outcomes related to perinatal mental health in South Asian immigrant women will be included. Healthcare provider and staff perspectives will be included only when findings specifically address perinatal mental health, care access, service delivery, barriers, facilitators, or support needs for South Asian immigrant women in Canada.

### Context

The context for this review is studies conducted in Canada and focusing on the perinatal period (pregnancy to one year postpartum). The perinatal period refers to the time from the onset of pregnancy through 12 months following childbirth. This period includes the antenatal (or prenatal) stage, meaning before birth, and the postnatal (or postpartum) stage, meaning after birth. The term perinatal is commonly used interchangeably with antenatal/prenatal and postnatal/postpartum in both clinical practice and research [17] the perinatal period is characterised by significant physical, emotional, and social changes, during which individuals may experience a wide range of emotions and an increased vulnerability to mental health difficulties [15]. Studies whose data are drawn from other countries, or which focus on non-perinatal periods, will be excluded. The review will begin in July 2026 and end in January 2027. At the time of manuscript submission, database searching and study selection have not yet been completed.

### Types of sources

This scoping review will include peer-reviewed empirical studies using qualitative, quantitative, or mixed-methods designs. Eligible qualitative designs may include, but are not limited to, phenomenology, grounded theory, ethnography, qualitative description, feminist research, action research, and community-based participatory research. Eligible quantitative studies may include observational, descriptive, and interventional designs, if they report outcomes relevant to perinatal mental health among South Asian immigrant women in Canada.

Relevant grey literature will also be considered, including Public Health Agency of Canada, Statistics Canada, Immigration, Refugees and Citizenship Canada, provincial public health agencies, Canadian Mental Health Association, Centre for Addiction and Mental Health, South Asian community organizations, settlement agency websites, and relevant theses/dissertation repositories., where sufficient information is available to determine eligibility and extract key charting items. Protocols of ongoing or planned studies will be included to identify emerging evidence and will be charted separately from completed studies because they do not report completed findings.

Review articles, including systematic and scoping reviews, will not be included as evidence sources in the main analysis; however, their reference lists will be screened to identify additional eligible primary studies. Editorials, commentaries, opinion pieces, letters, blogs, and narrative articles without original data or a study plan will be excluded.

### Information sources and search strategy

Searches will be conducted across key health and social science databases, including MEDLINE, Embase, PsycINFO, CINAHL, and Scopus. These databases were selected because, collectively, they cover research from medicine, nursing and allied health, psychology, mental health, and the social sciences. The search strategy will be developed in consultation with a health sciences librarian and will follow Joanna Briggs Institute guidance for scoping reviews. Initially, a limited search of two databases, such as Ovid MEDLINE (S1 File) and Embase, will be conducted to refine search terms and identify relevant index headings. Keywords and subject headings will then be adapted for each database to maximize retrieval of relevant sources.

The search strategy will include terms related to five core concepts: the perinatal period, mental health, South Asian identity or country of origin, immigration or newcomer status, and the Canadian context. To ensure that community and experiential perspectives are not overlooked, grey literature will be identified through relevant Public Health Agency of Canada, Statistics Canada, Immigration, Refugees and Citizenship Canada, provincial public health agencies, Canadian Mental Health Association, Centre for Addiction and Mental Health, South Asian community organizations, settlement agency websites, and relevant theses/dissertation repositories. Reference lists of included sources and relevant reviews will also be screened to identify additional eligible primary studies.

Sources published from January 2000 to the date of the final search will be included. This date range was selected because South Asian immigration to Canada increased markedly from the early 2000s onward and has continued to grow in subsequent decades; therefore, literature from this period is more likely to reflect contemporary migration patterns, settlement contexts, diagnostic frameworks, and perinatal mental health service practices relevant to Canada [21]. Records with English titles and/or abstracts will be screened. Full-text sources will be included only if an English version is available. Non-English full texts without available translation support will be excluded. We acknowledge that this may introduce language bias and potentially limit identification of relevant evidence published in languages other than English. This limitation will be considered when interpreting the findings. A health sciences librarian will review the search strategy before database searches are conducted, and the full search strategy for at least one database will be provided as a supplementary file. Any changes to the search strategy during the review process will be documented and reported in the completed scoping review.

### Study selection

Following the search, all identified citations will be collated and uploaded into Covidence, a web-based systematic review management platform [22]. Duplicate records will be identified and removed within Covidence prior to screening. Study selection will be conducted in two stages, Step 1 will involve review title and abstract screening by two independent reviewers (B.H., T.K.) and step 2 full text review will be done by (B.H). As this review forms part of a doctoral research program, the full-text eligibility assessment will be undertaken by the doctoral researcher to maintain consistency in the application of eligibility criteria and decision-making across studies. To enhance methodological rigor, uncertainties regarding study eligibility will be discussed with the supervisory team and, where necessary, consultation will be sought from a subject expert or health sciences librarian. Eligibility decisions, including reasons for exclusion at the full-text stage, will be documented and maintained in an audit trail to support transparency and reproducibility. Prior to screening, both reviewers will participate in an initial calibration and training session to ensure shared understanding of the eligibility criteria. In the first stage, titles and abstracts will be screened independently by the two reviewers against the predefined inclusion and exclusion criteria. Before commencing full screening, a pilot test will be conducted in which both reviewers will screen a random sample of 25 titles and abstracts to assess consistency and clarity. The reviewers will then meet to discuss discrepancies, refine the inclusion and exclusion criteria if necessary, and ensure a shared approach to screening. Full title and abstract screening will proceed. In the second stage, full-text articles of potentially relevant sources will be retrieved and assessed by the B.H. for eligibility. Uncertainty about eligibility may be resolved by consulting a subject expert or librarian. Reasons for exclusion of sources at the full-text stage will be documented and reported. A table listing excluded full-text studies and reasons for exclusion will be included as a supplemental file in the final review. The overall study selection process including identification, screening, eligibility, and inclusion of sources will be presented using a PRISMA-ScR flow diagram [16]. Language restrictions will apply such that only studies with English abstracts will be screened for eligibility, with data extraction limited to full-text articles available in English, due to resource constraints.

### Data extraction

A tailored data extraction form will be developed by the primary reviewer, B.H., based on relevant literature, the review objectives, and studies identified during search strategy development. The data-charting form will be pilot tested by B.H. on two eligible studies to assess clarity, completeness, and usability in capturing the prespecified data items. The pilot-tested form will be reviewed with the supervisory team, and revisions will be made through team discussion and consensus. As this scoping review forms part of a doctoral research project, data extraction will be conducted by the primary reviewer (B.H.) to ensure consistency in the application of the charting framework across all included studies. Prior to full data extraction, the charting form will be pilot tested on eligible studies and reviewed with the supervisory team to assess clarity, completeness, and consistency in capturing the prespecified data items. Any uncertainties encountered during extraction will be discussed with the supervisory team and resolved through consensus. Modifications to the extraction framework will be documented and reported in the final review to enhance transparency and methodological rigor. An audit trail of extraction decisions and revisions to the charting framework will be maintained throughout the review process. The extraction framework will include: study identifiers (title, authors, year, country, study type, objectives); focus within perinatal care (antenatal, intrapartum, postnatal); participant characteristics (demographics and inclusion criteria); methodological details (design, data collection, analysis); contextual factors (Canadian region, healthcare setting, policy context); maternal health outcomes; participants’ perceptions and experiences related to the quality of perinatal care; barriers to accessing services; and any interventions or support initiatives for South Asian immigrant women and perinatal mental health–related risk factors and protective factors for South Asian immigrant women (e.g., social support/isolation, IPV, acculturative stress, discrimination/racism, language barriers, financial insecurity, immigration-related stressors, stigma/cultural expectations, prior mental health history/trauma, birth complications, and access to culturally safe care). Any changes to the extraction form during full review will be documented and reported in the final manuscript. Articles not available through Western University Libraries will be requested via the Interlibrary Loan (ILL) service.

### Data Analysis and Presentation

The data extracted from the included studies will be synthesized using a narrative, descriptive approach in line with recognized scoping review methodology. First, B.H., as the primary reviewer, will chart and summarize study characteristics using frequencies and descriptive statistics (where appropriate), including study design, objectives, geographic setting within Canada (province/region), healthcare setting, participant characteristics (demographic profile and immigration-related variables where reported), inclusion criteria, sampling strategy, sample size, data collection methods, analytic approach, policy/program context, and outcomes related to perinatal mental health. Results of this mapping will be presented in summary tables. Consistent with the nature of scoping reviews, no formal critical appraisal of individual studies will be conducted. Although studies will not be excluded based on methodological quality, study design characteristics, reported limitations, and contextual considerations will be charted where available to support interpretation of the evidence base and identify areas requiring future investigation.

Second, we will synthesize findings relevant to the review question using a descriptive content analysis of extracted findings (e.g., participant-reported experiences and authors’ interpretations). To maintain the independent nature of the doctoral work, B.H. will code the extracted data and iteratively refine the coding framework through consultation with the supervisory team. To promote consistency and reliability, an initial coding framework will be developed and applied to a sample of extracted studies by two reviewers independently. Coding decisions will be compared and discussed to refine code definitions and ensure a shared understanding of categories. Regular meetings will be held throughout the analysis process to review coding decisions, resolve discrepancies, and document refinements to the coding framework. An audit trail of coding decisions and framework revisions will be maintained to enhance transparency and methodological rigor. This approach will be used to identify patterns, recurring categories, and areas of divergence across the included sources. Reviewers will independently code the extracted data and iteratively refine a coding framework through comparison and discussion to identify patterns, recurring themes, and areas of divergence.

As an initial organizing framework, evidence will be charted across the following domains: perinatal mental health outcomes and definitions or measurement; risk and protective factors; service use and help-seeking; barriers and facilitators to accessing perinatal and mental health services; support systems and coping strategies; maternal outcomes; and interventions or support initiatives. This framework may be refined iteratively as analysis proceeds.

Final categorizations and groupings will be reviewed through team discussion to enhance consistency and transparency. The findings will be presented narratively and in summary tables to map the scope and nature of research in this area, including study characteristics, key categories, and gaps in the literature. This synthesis will help identify patterns across study types, highlight barriers, needs, and supports relevant to South Asian immigrant women in Canada, and inform directions for future research, policy, and practice.

## Discussion

Perinatal mental health concerns, including depression, anxiety, and stress, can affect maternal functioning, infant outcomes, and family well-being. Among South Asian women who have migrated to Canada and experience pregnancy or postpartum in Canada, mental health risks, protective factors, and access to support may be influenced by settlement-related stressors, changing family roles, language and system-navigation challenges, stigma, social support, and access to culturally safe care. However, Canadian evidence on this population is dispersed across disciplines and publication types, and it remains unclear which outcomes, determinants, assessment tools, services, and interventions have been studied.

This scoping review will map Canadian evidence on perinatal mental health among South Asian immigrant women from pregnancy to 12 months postpartum, including peer-reviewed studies, grey literature, and protocol papers. Protocol papers will be included to identify ongoing or emerging research, although they may not contribute outcome data. Unlike previous reviews that have focused primarily on immigrant populations broadly or on postpartum depression specifically, this review will comprehensively examine the full perinatal period and a broader range of mental health outcomes, while focusing specifically on South Asian immigrant women within the Canadian context. The review will also examine how South Asian immigrant women are defined and represented within the literature and whether important contextual characteristics are reported and analyzed. We expect to identify the outcomes and measures used, reported risk and protective factors, patterns of help-seeking and service use, and interventions/programs or care models evaluated or under development in Canadian settings, where available, as well as key gaps such as limited subgroup analyses by country of origin, language, religion, migration pathway, or immigration status, and inconsistent reporting of immigration-related and sociodemographic characteristics. Given the substantial diversity within South Asian populations, the review will also explore the extent to which studies account for heterogeneity across cultural, linguistic, religious, socioeconomic, and migration-related backgrounds. Particular attention will be given to whether factors such as migration history, length of residence in Canada, refugee or immigration status, family composition, employment conditions, income, and social support networks are considered in existing research. Mapping these contextual influences is expected to provide a more nuanced understanding of how perinatal mental health experiences may differ across South Asian immigrant communities.

Anticipated outputs include an evidence map to guide future research and practical insights for clinicians, service planners, policymakers, and community organizations to strengthen culturally safe, accessible perinatal mental health supports. Because this is a protocol, no conclusions can yet be drawn regarding the nature or direction of the evidence. Rather, the review is expected to identify areas where evidence is concentrated, where important gaps persist, and where future research may be needed to better understand the perinatal mental health experiences of South Asian immigrant women in Canada. Ethics approval is not required as no primary data will be collected. Findings will be disseminated through peer-reviewed publication and conference presentations. Anticipated limitations include heterogeneity in outcome definitions and measurement tools, variability in the level of detail and accessibility of some grey literature sources, and possible underreporting of South Asian subgroup-specific findings within broader immigrant or racialized population studies. In addition, as this is a scoping review, the purpose is to map the extent and nature of the evidence rather than to assess intervention effectiveness. The findings may have important implications for policy and practice by identifying populations, service gaps, and contextual factors that are currently underrepresented within Canadian perinatal mental health research. This may support the development of more culturally responsive screening approaches, tailored mental health services, community-based supports, and policies that recognize the diversity of South Asian immigrant communities. Furthermore, identifying gaps in reporting related to migration history, socioeconomic status, language, and cultural background may help inform future research standards and improve the quality and applicability of evidence in this field. Nonetheless, synthesizing the available Canadian evidence, including ongoing work captured through protocols, will provide a foundation for targeted research, policy, and practice improvements.

## Supporting Information

S1 File MEDLINE search strategy. (ZIP)

S1 Checklist PRISMA-P checklist. (DOCX)
