# Supplementary material for: Perinatal mental health among South Asian immigrant women in Canada: A scoping review protocol
Source: PLoS One. 2026 Jul 20;21(7):e0354152. doi: 10.1371/journal.pone.0354152 (PMC13384276; doi:10.1371/journal.pone.0354152)
Supplement: S1 File — (ZIP) [file pone.0354152.s001.zip › S1. File.docx]

**S1. File. Search Strategy**

**Ovid Medline**

| **S.No** | **Keywords** | **Display** |
| --- | --- | --- |
| 1 | ("mental health" or depression or anxiety or postpartum).mp. or Mental Health/ or Depression/ or Depression, Postpartum/ [mp=title, book title, abstract, original title, name of substance word, subject heading word, floating sub-heading word, keyword heading word, organism supplementary concept word, protocol supplementary concept word, rare disease supplementary concept word, unique identifier, synonyms, population supplementary concept word, anatomy supplementary concept word] | 1124007 |
| 2 | (prenatal or postnatal or antenatal or postpartum or perinatal or pregnan* or "after birth" or after-birth).mp. or exp Perinatal Care/ or Pregnancy Maintenance/ or Labor, Obstetric/ [mp=title, book title, abstract, original title, name of substance word, subject heading word, floating sub-heading word, keyword heading word, organism supplementary concept word, protocol supplementary concept word, rare disease supplementary concept word, unique identifier, synonyms, population supplementary concept word, anatomy supplementary concept word] | 1437696 |
| 3 | (immigrant* or immigration or newcomer* or "south Asian*" or Asian* or India* or Pakistan* or "Sri Lanka*" or Bangladesh* or Emigrants Immigrants exp Asia, Southern or Asian People).mp. Bhutan* or Maldives* or Nepal* [mp=title, book title, abstract, original title, name of substance word, subject heading word, floating sub-heading word, keyword heading word, organism supplementary concept word, protocol supplementary concept word, rare disease supplementary concept word, unique identifier, synonyms, population supplementary concept word, anatomy supplementary concept word] | 673309 |
| 4 | South Asian identity/country of origin terms: exp South Asia/ or exp India/ or exp Pakistan/ or exp Bangladesh/ or exp Sri Lanka/ or exp Nepal/ or exp Bhutan/ or exp Maldives/ or exp Afghanistan/ or ("South Asian*" or "South-Asian*" or India* or Indian* or Pakistan* or Pakistani* or Bangladesh* or Bangladeshi* or "Sri Lanka*" or "Sri Lankan*" or Nepal* or Nepali* or Nepalese* or Bhutan* or Bhutanese* or Maldives or Maldivian* or Afghan* or Afghanistan* or Punjabi* or Panjabi* or Hindi or Urdu or Tamil* or Bengali* or Bangla or Sikh* or Hindu*).mp. | 46321 |
| 5 | (Canad* or Ontario or British Columbia or Quebec or Toronto or Vancouver or Montreal or Ottawa).mp. [mp=title, book title, abstract, original title, name of substance word, subject heading word, floating sub-heading word, keyword heading word, organism supplementary concept word, protocol supplementary concept word, rare disease supplementary concept word, unique identifier, synonyms, population supplementary concept word, anatomy supplementary concept word] | 326014 |
| 6 | 1 and 2 and 3 and 4 and 5 | 212 |
