# Supplementary material for: Perinatal mental health among South Asian immigrant women in Canada: A scoping review protocol
Source: PLoS One. 2026 Jul 20;21(7):e0354152. doi: 10.1371/journal.pone.0354152 (PMC13384276; doi:10.1371/journal.pone.0354152)
Supplement: Attachment — Submitted filename: Response to Reviewers.docx [file pone.0354152.s003.docx]

**Response to Reviewers**

Manuscript Title:
Perinatal Mental Health Among South Asian Immigrant Women in Canada: A Scoping Review Protocol

Dear Academic Editor and Reviewer,

We sincerely thank you for your careful review of our manuscript and for the constructive feedback provided. We appreciate the opportunity to revise our protocol and have addressed all comments accordingly. Below, we provide a point-by-point response outlining the revisions made in the manuscript.

| **Reviewer comments** | **Response to reviewer** |
| --- | --- |
| **Abstract** |  |
| Clarification of the Review Focus and Questions | We expanded the description of the review objectives to specify that the review will examine reported perinatal mental health outcomes, associated risk and protective factors, help-seeking experiences, and barriers and facilitators to accessing support services among South Asian immigrant women in Canada. This revision provides greater specificity regarding the scope and focus of the review. |
| Justification for Focusing on South Asian Immigrant Women | We added a statement explaining that South Asian women represent one of the largest and fastest-growing immigrant populations in Canada and may experience unique cultural, familial, linguistic, and migration-related influences that shape perinatal mental health experiences and access to care. This addition strengthens the rationale for focusing specifically on this population. |
| Reporting and Formatting Corrections | The sentence now reads: "This protocol was developed in accordance with Joanna Briggs Institute guidance for scoping review protocols and reported using the PRISMA-P checklist, where applicable (see S2 Checklist). |
| Additional Detail Regarding Eligibility Criteria | We added a brief description of the eligible population, specifying that the review will include immigrant women of South Asian origin (e.g. India, Pakistan, Bangladesh, Sri Lanka, Afghanistan, Nepal, Bhutan, and Maldives, backgrounds) residing in Canada during the perinatal period. This revision improves transparency regarding participant eligibility. |
| Clarification of the Evidence Quality/Appraisal Approach | We added a statement explaining that, consistent with scoping review methodology, studies will not be excluded based on methodological quality. We further clarified that study characteristics and reported methodological limitations will be charted to support interpretation of the evidence base. |
| Expanded Description of the Anticipated Contribution | We added a concluding statement indicating that the review will provide a comprehensive overview of the existing evidence and identify knowledge gaps to support culturally responsive perinatal mental health research, policy development, and healthcare practice for South Asian immigrant women in Canada. This addition clarifies the expected significance and contribution of the review. |
| **Introduction** |  |
| The introduction lacks a more robust theoretical or conceptual framework linking immigration, culture, and perinatal mental health. | Thank you for this suggestion. We added a new paragraph introducing a conceptual perspective that describes migration as a social and cultural transition involving acculturation, identity negotiation, social integration, and adaptation processes. We further explain how these factors intersect with motherhood, family expectations, and healthcare experiences to shape perinatal mental health outcomes among immigrant women. |
| The justification for focusing on South Asian immigrant women should be explored more thoroughly and should emphasize unique cultural and contextual factors rather than relying primarily on prevalence statistics. | We expanded the rationale for focusing on South Asian immigrant women by describing culturally specific influences such as collectivist family structures, gender norms, intergenerational dynamics, culturally shaped understandings of mental health, stigma, and help-seeking practices. These additions provide a stronger contextual justification for studying this population. |
| There is a lack of synthesis of previous research, with limited discussion of methodological limitations, inconsistencies, and knowledge gaps. | We added a synthesis paragraph highlighting fragmentation within the existing literature, variation in study designs, populations, outcomes, and assessment methods, and the tendency to aggregate immigrant populations. We also identified gaps related to underrepresentation of South Asian women and limited attention to mental health outcomes beyond postpartum depression. |
| The opening sentence beginning “While previous systematic and scoping reviews…” is overly long and repetitive. | We revised this section by separating the introductory statement from the discussion of Khan’s review, improving readability and reducing repetition while retaining the original content and rationale. |
| The contribution, novelty, and research gap of the proposed scoping review should be stated more clearly and succinctly. | We strengthened the final paragraph by explicitly stating that no recent scoping review has comprehensively mapped Canadian evidence across the full perinatal period while focusing specifically on South Asian immigrant women. We also clarified the review’s contribution as providing an updated evidence map to inform future research, policy, and culturally responsive practice. |
| **Methods** |  |
| The review question is broad and would benefit from more precise operational definitions of "South Asian immigrant women" and "perinatal mental health." | Thank you for this suggestion. We have added explicit operational definitions of both terms within the Review Question and Eligibility Criteria sections. South Asian immigrant women are now clearly defined according to country of origin and migration status, while perinatal mental health is defined as mental health outcomes occurring during pregnancy and up to 12 months postpartum, including depression, anxiety, stress, trauma, psychological distress, coping, resilience, and emotional wellbeing. |
| Including a variety of immigrant categories may result in significant heterogeneity that needs to be examined analytically. | We agree and have added text acknowledging the diversity of immigration pathways represented within the review population. We now specify that immigration-related variables such as refugee status, family sponsorship, economic migration, temporary residence, and length of residence in Canada will be charted and reported separately where available to facilitate interpretation of heterogeneity across studies. |
| Full-text screening appears to be conducted primarily by one reviewer, raising concerns regarding selection bias. | Thank you for this important observation. We acknowledge that full-text screening will be conducted by the primary reviewer as part of a doctoral research project. To clarify this process and enhance transparency, we have revised the manuscript to explicitly state that title and abstract screening will be conducted independently by two reviewers following calibration exercises. Full-text review will be undertaken by the doctoral researcher to ensure consistency in the application of eligibility criteria across studies. To mitigate potential selection bias, uncertainties regarding eligibility will be discussed with the supervisory team and, where required, consultation will be sought from a subject expert or health sciences librarian. In addition, all exclusion decisions at the full-text stage will be documented and reported, and an audit trail will be maintained throughout the review process. We have acknowledged this approach as a potential limitation while outlining measures taken to support rigor and transparency. |
| Data extraction by a single reviewer may jeopardize reliability. Independent duplicate extraction is advised. | Thank you for this important observation. We acknowledge that data extraction will be conducted by the primary reviewer as part of a doctoral research project. To improve transparency and methodological rigor, we have revised the manuscript to clarify the procedures used to support consistency and reliability. Specifically, the data extraction form will be pilot tested prior to full extraction, reviewed with the supervisory team, and refined through discussion and consensus. Any uncertainties encountered during extraction will be discussed with the supervisory team, and modifications to the charting framework will be documented. An audit trail of extraction decisions and revisions will also be maintained throughout the review process. We have acknowledged that single-reviewer extraction may represent a limitation of the review and have outlined the measures implemented to minimize potential extraction errors and enhance consistency. |
| Excluding non-English full texts may introduce language bias. | We acknowledge this limitation and have revised the manuscript to explicitly recognize the potential for language bias resulting from exclusion of non-English full texts. The rationale for this decision, namely resource and translation constraints, has been clarified and the limitation will be considered when interpreting review findings. |
| More specificity is needed regarding procedures for ensuring consistency and reliability in content analysis. | We expanded the Data Analysis section to describe the use of an initial coding framework, independent coding of a sample of studies, regular reviewer meetings, consensus discussions, iterative refinement of codes, and maintenance of an audit trail to enhance consistency, transparency, and reliability in content analysis. |
| **Discussion** |  |
| The discussion reads more like a protocol justification than an interpretation of the results and is mostly conjectural. It does not sufficiently distinguish between expected outcomes and conclusions derived from the evidence. | Thank you for this observation. We have revised the Discussion to more clearly distinguish anticipated findings from actual review findings. We explicitly state that, as this is a protocol, no conclusions can yet be drawn regarding the nature or direction of the evidence and that the discussion focuses on the anticipated contribution of the review and expected areas of inquiry. |
| The authors should provide a more thorough explanation of expected knowledge gaps and outline how the review will go beyond earlier perinatal mental health reviews. | We strengthened the Discussion by clarifying how the proposed review differs from previous reviews, including its focus on the full perinatal period, broader mental health outcomes, inclusion of grey literature and protocols, and specific attention to South Asian immigrant women within the Canadian context. We also expanded discussion of anticipated knowledge gaps related to subgroup representation, immigration-related variables, and contextual determinants. |
| The diversity within South Asian communities and the effects of contextual elements such as socioeconomic status and migration history should receive more attention. | We expanded the Discussion to emphasize the heterogeneity of South Asian populations and the importance of examining factors such as migration history, immigration status, length of residence in Canada, language, religion, socioeconomic status, family structure, employment conditions, and social support networks. |
| Practical and policy implications require more critical elaboration and connection to the expected results of the evidence synthesis. | We revised the Discussion to provide a more detailed explanation of how the anticipated findings may inform culturally responsive screening, service delivery, community-based supports, policy development, and future research priorities. We also linked these implications to expected evidence gaps and patterns identified through the scoping review. |
| **Reference Style** |  |
| Authors are recommended to check the correct reference styles following the journal’s guidelines. | Thank you for this valuable comment. We carefully reviewed the reference list and revised it to ensure consistency with the PLOS ONE reference style guidelines. Specifically, we standardized the formatting of journal articles, reports, dissertations, and web-based resources according to Vancouver style, corrected author and journal formatting where required, added missing publication details and access information for online sources, and ensured consistency in DOI presentation across all references. These revisions have been incorporated throughout the manuscript. |
