# Supplementary material for: Perinatal mental health among South Asian immigrant women in Canada: A scoping review protocol
Source: PLoS One. 2026 Jul 20;21(7):e0354152. doi: 10.1371/journal.pone.0354152 (PMC13384276; doi:10.1371/journal.pone.0354152)
Supplement: Peer review history

# Peer review: Decision Letter 0

**DOI:** 10.1371/journal.pone.0354152.r001

**Authors:** Nicolas Padilla-Raygoza

19 Jun 2026

PONE-D-26-22899

Perinatal mental health among South Asian immigrant women in Canada: A scoping review protocol

PLOS One

Dear Dr. Hajira,

Thank you for submitting your manuscript to PLOS ONE. After careful consideration, we feel that it has merit but does not fully meet PLOS ONE’s publication criteria as it currently stands. Therefore, we invite you to submit a revised version of the manuscript that addresses the points raised during the review process.

I consider that authors should include answers to the reviewers' comments.

Please submit your revised manuscript by Jul 04 2026 11:59PM. If you will need more time than this to complete your revisions, please reply to this message or contact the journal office at plosone@plos.org. When you're ready to submit your revision, log on to https://www.editorialmanager.com/pone/ and select the 'Submissions Needing Revision' folder to locate your manuscript file.

Please include the following items when submitting your revised manuscript:

- A letter that responds to each point raised by the academic editor and reviewer(s). You should upload this letter as a separate file labeled 'Response to Reviewers'.
- A marked-up copy of your manuscript that highlights changes made to the original version. You should upload this as a separate file labeled 'Revised Manuscript with Track Changes'.
- An unmarked version of your revised paper without tracked changes. You should upload this as a separate file labeled 'Manuscript'.

If you would like to make changes to your financial disclosure, please include your updated statement in your cover letter. Guidelines for resubmitting your figure files are available below the reviewer comments at the end of this letter.

If applicable, we recommend that you deposit your laboratory protocols in protocols.io to enhance the reproducibility of your results. Protocols.io assigns your protocol its own identifier (DOI) so that it can be cited independently in the future. For instructions see: https://journals.plos.org/plosone/s/submission-guidelines#loc-laboratory-protocols. Additionally, PLOS ONE offers an option for publishing peer-reviewed Lab Protocol articles, which describe protocols hosted on protocols.io. Read more information on sharing protocols at https://plos.org/protocols?utm_medium=editorial-email&utm_source=authorletters&utm_campaign=protocols.

As the corresponding author, your ORCID iD is verified in the submission system and will appear in the published article. PLOS supports the use of ORCID, and we encourage all coauthors to register for an ORCID iD and use it as well. Please encourage your coauthors to verify their ORCID iD within the submission system before final acceptance, as unverified ORCID iDs will not appear in the published article. *Only*  the individual author can complete the verification step; PLOS staff *cannot*  verify ORCID iDs on behalf of authors.

We look forward to receiving your revised manuscript.

Kind regards,

Nicolas Padilla-Raygoza, Doctorate in Sciences

Academic Editor

PLOS One

Journal Requirements:

When submitting your revision, we need you to address these additional requirements.

1. Please ensure that your manuscript meets PLOS ONE's style requirements, including those for file naming. The PLOS ONE style templates can be found at

https://journals.plos.org/plosone/s/file?id=wjVg/PLOSOne_formatting_sample_main_body.pdf and

https://journals.plos.org/plosone/s/file?id=ba62/PLOSOne_formatting_sample_title_authors_affiliations.pdf

2. When completing the data availability statement of the submission form, you indicated that you will make your data available on acceptance. We strongly recommend all authors decide on a data sharing plan before acceptance, as the process can be lengthy and hold up publication timelines. Please note that, though access restrictions are acceptable now, your entire data will need to be made freely accessible if your manuscript is accepted for publication. This policy applies to all data except where public deposition would breach compliance with the protocol approved by your research ethics board. If you are unable to adhere to our open data policy, please kindly revise your statement to explain your reasoning and we will seek the editor's input on an exemption. Please be assured that, once you have provided your new statement, the assessment of your exemption will not hold up the peer review process.

3. Please include captions for your Supporting Information files at the end of your manuscript, and update any in-text citations to match accordingly. Please see our Supporting Information guidelines for more information: http://journals.plos.org/plosone/s/supporting-information.

4. If the reviewer comments include a recommendation to cite specific previously published works, please review and evaluate these publications to determine whether they are relevant and should be cited. There is no requirement to cite these works unless the editor has indicated otherwise.

5. Please review your reference list to ensure that it is complete and correct. If you have cited papers that have been retracted, please include the rationale for doing so in the manuscript text, or remove these references and replace them with relevant current references. Any changes to the reference list should be mentioned in the rebuttal letter that accompanies your revised manuscript. If you need to cite a retracted article, indicate the article’s retracted status in the References list and also include a citation and full reference for the retraction notice.

Additional Editor Comments:

The authors should take into account the reviewer's comments and resubmit the manuscript. I consider that answered the comments from the reviewer, the protocol will be better.

[Note: HTML markup is below. Please do not edit.]

Reviewers' comments:

Reviewer's Responses to Questions

**Comments to the Author**

1. Does the manuscript provide a valid rationale for the proposed study, with clearly identified and justified research questions?

The research question outlined is expected to address a valid academic problem or topic and contribute to the base of knowledge in the field.

Reviewer #1: Yes

**********

2. Is the protocol technically sound and planned in a manner that will lead to a meaningful outcome and allow testing the stated hypotheses?

The manuscript should describe the methods in sufficient detail to prevent undisclosed flexibility in the experimental procedure or analysis pipeline, including sufficient outcome-neutral conditions (e.g. necessary controls, absence of floor or ceiling effects) to test the proposed hypotheses and a statistical power analysis where applicable. As there may be aspects of the methodology and analysis which can only be refined once the work is undertaken, authors should outline potential assumptions and explicitly describe what aspects of the proposed analyses, if any, are exploratory.

Reviewer #1: Yes

**********

3. Is the methodology feasible and described in sufficient detail to allow the work to be replicable?

Descriptions of methods and materials in the protocol should be reported in sufficient detail for another researcher to reproduce all experiments and analyses. The protocol should describe the appropriate controls, sample size calculations, and replication needed to ensure that the data are robust and reproducible.

Reviewer #1: Yes

**********

4. Have the authors described where all data underlying the findings will be made available when the study is complete?

The PLOS Data policy requires authors to make all data underlying the findings described in their manuscript fully available without restriction, with rare exception, at the time of publication. The data should be provided as part of the manuscript or its supporting information, or deposited to a public repository. For example, in addition to summary statistics, the data points behind means, medians and variance measures should be available. If there are restrictions on publicly sharing data—e.g. participant privacy or use of data from a third party—those must be specified.

Reviewer #1: Yes

**********

5. Is the manuscript presented in an intelligible fashion and written in standard English?

PLOS ONE does not copyedit accepted manuscripts, so the language in submitted articles must be clear, correct, and unambiguous. Any typographical or grammatical errors should be corrected at revision, so please note any specific errors here.

Reviewer #1: Yes

**********

6. Review Comments to the Author

Please use the space provided to explain your answers to the questions above and, if applicable, provide comments about issues authors must address before this protocol can be accepted for publication. You may also include additional comments for the author, including concerns about research or publication ethics.

You may also provide optional suggestions and comments to authors that they might find helpful in planning their study.

(Please upload your review as an attachment if it exceeds 20,000 characters)

Reviewer #1: To inform future research, practice, and policy, this scoping review will map the amount, variety, and type of evidence on perinatal mental health among South Asian immigrant women in Canada and identify gaps. My observations are given below:

(1) The study title is okay. The abstract needs minor improvements. More specificity would improve the abstract. Give a clear explanation of the review questions and the justification for concentrating on South Asian women. Address discrepancies in reporting (e.g. The g. PRISMA-P statement punctuation). Give a brief explanation of the anticipated contribution, the specifics of the eligibility requirements, and the strategy for evaluating the qualities of the evidence.

(2) The introduction tackles a significant and understudied subject, but there are a few things that could be done better. First, there is no more robust theoretical or conceptual framework connecting immigration, culture, and perinatal mental health in the literature review, which is primarily descriptive. Second, rather than mainly depending on prevalence statistics, the justification for concentrating on South Asian immigrant women needs to be more thoroughly examined, emphasizing distinctive cultural and contextual elements. Third, there is a lack of synthesis in the review of earlier research, with little attention paid to methodological flaws, knowledge gaps, and inconsistencies. The opening line, "While previous systematic and scoping reviews..." is excessively lengthy and repetitious. Lastly, the proposed scoping review's contribution, novelty, and research gap should be stated more succinctly and clearly.

(3) Although the methods section is generally thorough, there are a few methodological issues that need to be addressed. The review question is broad and would benefit from more precise operational definitions of "South Asian immigrant women" and "perinatal mental health.". Including a variety of immigrant categories may result in significant heterogeneity that needs to be examined analytically. Because full-text screening seems to be done mostly by one reviewer, which raises the possibility of selection bias, study selection lacks methodological rigor. In a similar vein, independent duplicate extraction is advised because data extraction by a single reviewer may jeopardize reliability. Language bias may be introduced by the justification for excluding full texts written in languages other than English. Lastly, more specificity is needed in the processes for guaranteeing consistency and reliability in content analysis.

(4) The discussion reads more like a protocol justification than an interpretation of the results and is mostly conjectural. It may mislead readers about the review's contribution because it does not sufficiently distinguish between expected outcomes and conclusions derived from the evidence. By giving a more thorough explanation of the expected knowledge gaps and outlining how the review will go beyond earlier perinatal mental health reviews, the authors should bolster the conversation. The diversity within South Asian communities and the effects of contextual elements like socioeconomic status and migration history should receive more attention. More critical elaboration and a connection to the expected results of the evidence synthesis are needed for the practical and policy implications.

(5) Authors are recommended to check the correct reference styles following the journal’s guidelines.

**********

7. PLOS authors have the option to publish the peer review history of their article (what does this mean?). If published, this will include your full peer review and any attached files.

If you choose “no”, your identity will remain anonymous but your review may still be made public.

**Do you want your identity to be public for this peer review?**  For information about this choice, including consent withdrawal, please see our Privacy Policy.

Reviewer #1: **Yes:**  Gyanesh Kumar Tiwari

**********

[NOTE: If reviewer comments were submitted as an attachment file, they will be attached to this email and accessible via the submission site. Please log into your account, locate the manuscript record, and check for the action link "View Attachments". If this link does not appear, there are no attachment files.]

To ensure your figures meet our technical requirements, please review our figure guidelines: https://journals.plos.org/plosone/s/figures

You may also use PLOS’s free figure tool, NAAS, to help you prepare publication quality figures: https://journals.plos.org/plosone/s/figures#loc-tools-for-figure-preparation.

NAAS will assess whether your figures meet our technical requirements by comparing each figure against our figure specifications.

# Author comment: Author response to Decision Letter 1

**DOI:** 10.1371/journal.pone.0354152.r002

30 Jun 2026

We sincerely thank the editor and reviewers for their careful evaluation of our manuscript and for their constructive comments. We have carefully considered all suggestions and revised the manuscript accordingly. The revisions have improved the clarity and quality of the manuscript. Detailed responses to each comment are provided below.

Abstract

Comment 1: Clarification of the Review Focus and Questions

Response: We expanded the description of the review objectives to specify that the review will examine reported perinatal mental health outcomes, associated risk and protective factors, help-seeking experiences, and barriers and facilitators to accessing support services among South Asian immigrant women in Canada. This revision provides greater specificity regarding the scope and focus of the review.

Comment 2: Justification for Focusing on South Asian Immigrant Women

Response: We added a statement explaining that South Asian women represent one of the largest and fastest-growing immigrant populations in Canada and may experience unique cultural, familial, linguistic, and migration-related influences that shape perinatal mental health experiences and access to care. This addition strengthens the rationale for focusing specifically on this population.

Comment 3: Reporting and Formatting Corrections

Response: The sentence now reads: This protocol was developed in accordance with Joanna Briggs Institute guidance for scoping review protocols and reported using the PRISMA-P checklist, where applicable (see S2 checklist).

Comment 4: Additional Detail Regarding Eligibility Criteria

Response: We added a brief description of the eligible population, specifying that the review will include immigrant women of South Asian origin (e.g., Indian, Pakistan, Bangladesh, Sri Lanka, Afghan, Nepal, Bhutan, and Maldives, backgrounds) residing in Canada during the perinatal period. This revision improves transparency regarding participant eligibility.

Comment 5: Clarification of the Evidence Quality/Appraisal Approach

Response: We added a statement explaining that, consistent with scoping review methodology, studies will not be excluded based on methodological quality. We further clarified that study characteristics and reported methodological limitations will be charted to support interpretation of the evidence base.

Comment 6: Expanded Description of the Anticipated Contribution

Response: We added a concluding statement indicating that the review will provide a comprehensive overview of the existing evidence and identify knowledge gaps to support culturally responsive perinatal mental health research, policy development, and healthcare practice for South Asian immigrant women in Canada. This addition clarifies the expected significance and contribution of the review.

Introduction

Comment 7: The introduction lacks a more robust theoretical or conceptual framework linking immigration, culture, and perinatal mental health.

Response: Thank you for this suggestion. We added a new paragraph introducing a conceptual perspective that describes migration as a social and cultural transition involving acculturation, identity negotiation, social integration, and adaptation processes. We further explain how these factors intersect with motherhood, family expectations, and healthcare experiences to shape perinatal mental health outcomes among immigrant women.

Comment 8: The justification for focusing on South Asian immigrant women should be explored more thoroughly and should emphasize unique cultural and contextual factors rather than relying primarily on prevalence statistics.

Response: We expanded the rationale for focusing on South Asian immigrant women by describing culturally specific influences such as collectivist family structures, gender norms, intergenerational dynamics, culturally shaped understandings of mental health, stigma, and help-seeking practices. These additions provide a stronger contextual justification for studying this population.

Comment 9: There is a lack of synthesis of previous research, with limited discussion of methodological limitations, inconsistencies, and knowledge gaps.

Response: We added a synthesis paragraph highlighting fragmentation within the existing literature, variation in study designs, populations, outcomes, and assessment methods, and the tendency to aggregate immigrant populations. We also identified gaps related to under-representation of South Asian women and limited attention to mental health outcomes beyond postpartum depression.

Comment 10: The opening sentence beginning “While previous systematic and scoping reviews…” is overly long and repetitive.

Response: We revised this section by separating the introductory statement from the discussion of Khan’s review, improving readability and reducing repetition while retaining the original content and rationale.

Comment 11: The contribution, novelty, and research gap of the proposed scoping review should be stated more clearly and succinctly.

Response: We strengthened the final paragraph by explicitly stating that no recent scoping review has comprehensively mapped Canadian evidence across the full perinatal period while focusing specifically on South Asian immigrant women. We also clarified the review’s contribution as providing an updated evidence map to inform future research, policy, and culturally responsive practice.

Methods

Comment 12: The review question is broad and would benefit from more precise operational definitions of "South Asian immigrant women" and "perinatal mental health".

Including a variety of immigrant categories may result in significant heterogeneity that needs to be examined analytically.

Response: Thank you for this suggestion. We have added explicit operational definitions of both terms within the Review Question and Eligibility Criteria sections. South Asian immigrant women are now clearly defined according to country of origin and migration status, while perinatal mental health is defined as mental health outcomes occurring during pregnancy and up to 12 months postpartum, including depression, anxiety, stress, trauma, psychological distress, coping, resilience, and emotional wellbeing.

We agree and have added text acknowledging the diversity of immigration pathways represented within the review population. We now specify that immigration-related variables such as refugee status, family sponsorship, economic migration, temporary residence, and length of residence in Canada will be charted and reported separately where available to facilitate interpretation of heterogeneity across studies.

Comment 13: Full-text screening appears to be conducted primarily by one reviewer, raising concerns regarding selection bias.

Response: Thank you for this important observation. We acknowledge that full-text screening will be conducted by the primary reviewer as part of a doctoral research project. To clarify this process and enhance transparency, we have revised the manuscript to explicitly state that title and abstract screening will be conducted independently by two reviewers following calibration exercises. Full-text review will be undertaken by the doctoral researcher to ensure consistency in the application of eligibility criteria across studies. To mitigate potential selection bias, uncertainties regarding eligibility will be discussed with the supervisory team and, where required, consultation will be sought from a subject expert or health sciences librarian. In addition, all exclusion decisions at the full-text stage will be documented and reported, and an audit trail will be maintained throughout the review process. We have acknowledged this approach as a potential limitation while outlining measures taken to support rigour and transparency.

Comment 14: Data extraction by a single reviewer may jeopardize reliability. Independent duplicate extraction is advised.

Response: Thank you for this important observation. We acknowledge that data extraction will be conducted by the primary reviewer as part of a doctoral research project. To improve transparency and methodological rigor, we have revised the manuscript to clarify the procedures used to support consistency and reliability. Specifically, the data extraction form will be pilot tested prior to full extraction, reviewed with the supervisory team, and refined through discussion and consensus. Any uncertainties encountered during extraction will be discussed with the supervisory team, and modifications to the charting framework will be documented. An audit trail of extraction decisions and revisions will also be maintained throughout the review process. We have acknowledged that single-reviewer extraction may represent a limitation of the review and have outlined the measures implemented to minimize potential extraction errors and enhance consistency.

Comment 15: Excluding non-English full texts may introduce language bias.

Response: We acknowledge this limitation and have revised the manuscript to explicitly recognize the potential for language bias resulting from exclusion of non-English full texts. The rationale for this decision, namely resource and translation constraints, has been clarified and the limitation will be considered when interpreting review findings.

Comment 16: More specificity is needed regarding procedures for ensuring consistency and reliability in content analysis.

Response: We expanded the Data Analysis section to describe the use of an initial coding framework, independent coding of a sample of studies, regular reviewer meetings, consensus discussions, iterative refinement of codes, and maintenance of an audit trail to enhance consistency, transparency, and reliability in content analysis.

Discussion

Comment 17: The discussion reads more like a protocol justification than an interpretation of the results and is mostly conjectural. It does not sufficiently distinguish between expected outcomes and conclusions derived from the evidence.

Response: Thank you for this observation. We have revised the Discussion to more clearly distinguish anticipated findings from actual review findings. We explicitly state that, as this is a protocol, no conclusions can yet be drawn regarding the nature or direction of the evidence and that the discussion focuses on the anticipated contribution of the review and expected areas of inquiry.

Comment 18: The authors should provide a more thorough explanation of expected knowledge gaps and outline how the review will go beyond earlier perinatal mental health reviews.

Response: We strengthened the discussion by clarifying how the proposed review differs from previous reviews, including its focus on the full perinatal period, broader mental health outcomes, inclusion of grey literature and protocols, and specific attention to South Asian immigrant women within the Canadian context. We also expanded discussion of anticipated knowledge gaps related to subgroup representation, immigration-related variables, and contextual determinants.

Comment 19: The diversity within South Asian communities and the effects of contextual elements such as socioeconomic status and migration history should receive more attention.

Response: We expanded the Discussion to emphasize the heterogeneity of South Asian populations and the importance of examining factors such as migration history, immigration status, length of residence in Canada, language, religion, socioeconomic status, family structure, employment conditions, and social support networks.

Comment 20: Practical and policy implications require more critical elaboration and connection to the expected results of the evidence synthesis.

Response: We revised the Discussion to provide a more detailed explanation of how the anticipated findings may inform culturally responsive screening, service delivery, community-based supports, policy development, and future research priorities. We also linked these implications to expected evidence gaps and patterns identified through the scoping review.

Reference Style

Comment 21: Authors are recommended to check the correct reference styles following the journal’s guidelines.

Response: Thank you for this valuable comment. We carefully reviewed the reference list and revised it to ensure consistency with the PLOS ONE reference style guidelines. Specifically, we standardized the formatting of journal articles, reports, dissertations, and web-based resources according to Vancouver style; corrected author and journal formatting where required; added missing publication details and access information for online sources; and ensured consistency in DOI presentation across all references. These revisions have been incorporated throughout the manuscript.

Attachment Submitted filename: Response to Reviewers.docx

# Editor report: Decision Letter 1

**DOI:** 10.1371/journal.pone.0354152.r003

**Authors:** Nicolas Padilla-Raygoza

6 Jul 2026

Perinatal mental health among South Asian immigrant women in Canada: A scoping review protocol

PONE-D-26-22899R1

Dear Dr. Hajira,

We’re pleased to inform you that your manuscript has been judged scientifically suitable for publication and will be formally accepted for publication once it meets all outstanding technical requirements.

Within one week, you’ll receive an e-mail detailing the required amendments. When these have been addressed, you’ll receive a formal acceptance letter and your manuscript will be scheduled for publication.

An invoice will be generated when your article is formally accepted. Please note, if your institution has a publishing partnership with PLOS and your article meets the relevant criteria, all or part of your publication costs will be covered. Please make sure your user information is up-to-date by logging into Editorial Manager at Editorial Manager® and clicking the ‘Update My Information' link at the top of the page. For questions related to billing, please contact billing support.

If your institution or institutions have a press office, please notify them about your upcoming paper to help maximize its impact. If they’ll be preparing press materials, please inform our press team as soon as possible -- no later than 48 hours after receiving the formal acceptance. Your manuscript will remain under strict press embargo until 2 pm Eastern Time on the date of publication. For more information, please contact onepress@plos.org.

Kind regards,

Nicolas Padilla-Raygoza, Doctorate in Sciences

Academic Editor

PLOS One

Additional Editor Comments (optional):

After review the new submision, the authors answered all comments from reviewer, the article has the requirements for publish in Plos One.

Reviewers' comments:

# Editor report: Acceptance letter

**DOI:** 10.1371/journal.pone.0354152.r004

**Authors:** Nicolas Padilla-Raygoza

PONE-D-26-22899R1

PLOS One

Dear Dr. Hajira,

I'm pleased to inform you that your manuscript has been deemed suitable for publication in PLOS One. Congratulations! Your manuscript is now being handed over to our production team.

At this stage, our production department will prepare your paper for publication. This includes ensuring the following:

* All references, tables, and figures are properly cited

* All relevant supporting information is included in the manuscript submission,

* There are no issues that prevent the paper from being properly typeset

You will receive further instructions from the production team, including instructions on how to review your proof when it is ready. Please keep in mind that we are working through a large volume of accepted articles, so please give us a few days to review your paper and let you know the next and final steps.

Lastly, if your institution or institutions have a press office, please let them know about your upcoming paper now to help maximize its impact. If they'll be preparing press materials, please inform our press team within the next 48 hours. Your manuscript will remain under strict press embargo until 2 pm Eastern Time on the date of publication. For more information, please contact onepress@plos.org.

You will receive an invoice from PLOS for your publication fee after your manuscript has reached the completed accept phase. If you receive an email requesting payment before acceptance or for any other service, this may be a phishing scheme. Learn how to identify phishing emails and protect your accounts at https://explore.plos.org/phishing.

If we can help with anything else, please email us at customercare@plos.org.

Thank you for submitting your work to PLOS ONE and supporting open access.

Kind regards,

PLOS ONE Editorial Office Staff

on behalf of

Dr. Nicolas Padilla-Raygoza

Academic Editor

PLOS One
